# Serum Calprotectin in Hospitalized Patients with COVID-19 in Relation to High-Dimensional Serum Proteomic Patterns

**DOI:** 10.3390/ijms27031243

**Published:** 2026-01-26

**Authors:** Åsa Parke, Benedikt Strunz, Puran Chen, Karolinska KI/K COVID-19 Study Group, Dorota Religa, Hans-Gustaf Ljunggren, Olav Rooyackers, Soo Aleman, Anna Norrby-Teglund, Niklas K. Björkström, Magnus Hansson, Kristoffer Strålin

**Affiliations:** 1 Department of Infectious Diseases, Karolinska University Hospital, 141 57 Stockholm, Sweden; soo.aleman@ki.se (S.A.); kristoffer.stralin@ki.se (K.S.); 2 Center for Infectious Medicine, Department of Medicine Huddinge, Karolinska Institutet, 141 57 Stockholm, Sweden; benedikt.struntz@ki.se (B.S.); puran.chen@ki.se (P.C.); hans-gustaf.ljunggren@ki.se (H.-G.L.); anna.norrby-teglund@ki.se (A.N.-T.); niklas.bjorkstrom@ki.se (N.K.B.); 3 Department of Inflammation and Aging, Karolinska University Hospital, 141 57 Stockholm, Sweden; dorota.religa@ki.se; 4 Department of Neurobiology, Care Sciences and Society (NVS), Karolinska Institutet, 171 77 Stockholm, Sweden; 5 Perioperative Medicine and Intensive Care, Karolinska University Hospital, 171 77 Stockholm, Sweden; olav.rooyackers@ki.se; 6 Department of Clinical Interventions and Technology, Karolinska Institutet, 171 77 Stockholm, Sweden; 7 Department of Clinical Chemistry, Karolinska University Hospital, 141 57 Stockholm, Sweden; magnus.d.hansson@regionstockholm.se; 8 Department of Laboratory Medicine, Division of Clinical Chemistry, Karolinska Institutet, 171 77 Stockholm, Sweden

**Keywords:** COVID-19, COVID-19 severity, sepsis, serum-calprotectin, S10012A, proteomics

## Abstract

Calprotectin in blood has been identified as a potential biomarker for severe COVID-19 and sepsis. As a knowledge gap remains regarding the biological role of calprotectin, we aimed to investigate the association between serum calprotectin and the circulating proteome in patients with COVID-19 as a model for viral sepsis. In this observational study, serum samples were collected from 160 hospitalized adult patients with COVID-19. The samples were analyzed for calprotectin using a routine turbidimetric assay and for proteomics using the Olink Explore 1536 platform. Patients were classified as having severe or moderate COVID-19 according to oxygen supply on the day of blood sampling. The median calprotectin level was significantly higher in patients with severe compared to moderate COVID-19. In relation to proteomics, calprotectin levels were associated with a neutrophil-centered inflammatory proteomic signature, characterized by upregulation of cytokine and danger-signaling pathways. S100A12 showed the strongest correlation to calprotectin. In conclusion, calprotectin is associated with disease severity in COVID-19, and high levels reflect a neutrophil-driven inflammatory proteomic profile, particularly involving S100A12. These findings support calprotectin as a biomarker of neutrophil-mediated hyperinflammation in viral sepsis.

## 1. Introduction

The antiviral immune response in COVID-19 and the induced hyperinflammatory phase in patients with severe disease have been widely studied but not yet fully understood [1,2,3,4,5,6]. A large proportion of hospitalized patients with COVID-19 in 2020 fulfilled the Sepsis-3 criteria [7] and constituted a relatively homogeneous sepsis population, with infection caused by a single microorganism and predominant organ dysfunction in the respiratory system [7,8,9]. Thus, COVID-19 during 2020 could be used as a model for viral sepsis in studies of sepsis pathogenesis [10].

The protein calprotectin is a heterodimer consisting of two subunits, S100A8 and S100A9 [11], and is present in the cytoplasm of neutrophils [12]. It is released into the circulation upon activation of neutrophils at inflammatory sites [13] and can trigger an immune response. It also exerts an antibacterial effect by restricting the availability of essential metal ions, particularly zinc and calcium [11].

Calprotectin in serum or plasma was early in the pandemic described as a potential biomarker for COVID-19 [14,15,16,17,18,19,20,21,22,23,24,25,26,27,28,29], as it was significantly elevated in patients with severe COVID-19 [30]. It has also been identified as a general sepsis biomarker. Our group [31] and others, for example, Huang et al. and Larsson et al., have found it to be an interesting marker for identifying clinical deterioration in patients with sepsis [31,32,33,34,35]. Calprotectin has also been found to be elevated in non-COVID viral respiratory infections, although at lower levels than in bacterial respiratory infections [36].

There remains a knowledge gap regarding the biological interpretation of serum calprotectin levels in COVID-19 and their relationship with the circulating proteome. It is unclear whether elevated calprotectin primarily reflects disease severity or whether it is associated with a distinct neutrophil-driven proteomic signature.

To address this knowledge gap, the present study aimed to investigate how serum calprotectin relates to the circulating proteome across COVID-19 disease severity and whether the proteomic signature associated with elevated calprotectin overlaps with that characterizing severe COVID-19.

## 2. Results

### 2.1. Study Cohort Characteristics

Of 257 SARS-CoV-2 positive hospitalized patients who had at least one serum sample available for analysis in the Karolinska KI/K COVID-19 biobank [37], 160 patients were included in the study as they were sampled within 8 days from hospital admission. Table 1 shows the characteristics of these patients. The median age was 59 years (interquartile range (IQR) 51–67 years), and 26% were female. The median Charlson Comorbidity Index (CCI) was 1. The diagnosis of hypertonia before hospitalization was the most common comorbidity, followed by diabetes. The maximum oxygen supply on the sample day was registered and used to define COVID-19 severity. A total of 109 patients (68%) had moderate COVID-19 (≤10 L/min supplementary oxygen), and 51 patients (32%) had severe COVID-19 (>10 L/min supplementary oxygen, high-flow nasal oxygen (HFNO), non-invasive ventilation (NIV), or invasive mechanical ventilation). Sepsis, according to Sepsis-3 criteria, was noted in 70% overall, i.e., 59% among those with moderate and 100% among those with severe COVID-19.

### 2.2. Serum Calprotectin in Relation to COVID-19 Severity

#### Serum Calprotectin and Disease Severity

**Figure 1:**
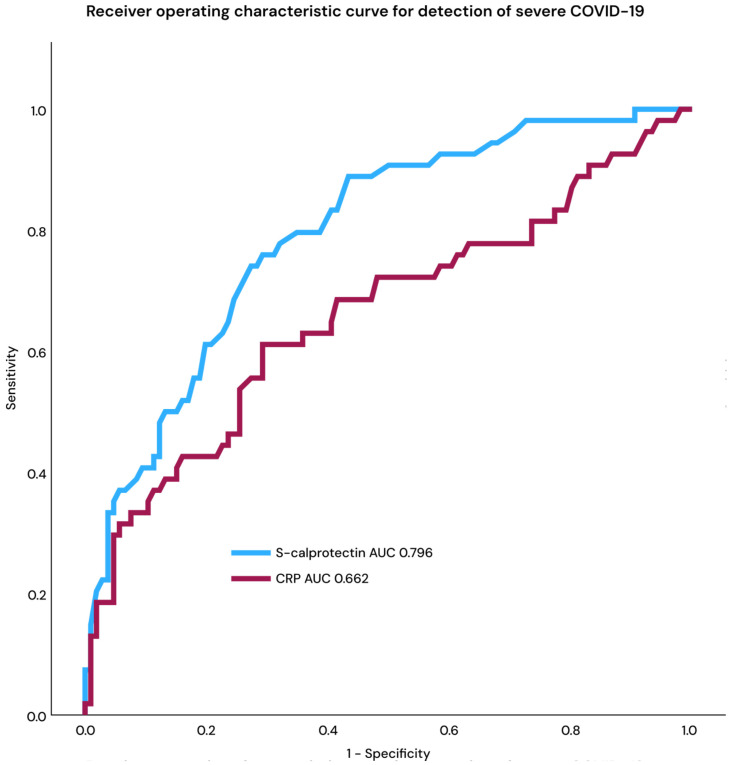
Receiver operating characteristic curve, s-calprotectin, and CRP for discrimination of severe from moderate COVID-19. Area under the curve and odds ratio (OR) and confidence interval (CI) were calculated for calprotectin compared to CRP (OR 1.35; 1.18–1.56).

The median calprotectin level for all study patients was 3.5 mg/L (IQR 2–5.6 mg/L); it was 5.4 mg/L (IQR 3.8–9.2 mg/L) for those with severe COVID-19 and 2.8 mg/L (IQR 1.6–4.1 mg/L) for those with moderate COVID-19 (*p* < 0.0001; Mann–Whitney U test). The median CRP for all patients was 94 mg/L (IQR 52–173); it was 80 mg/L (IQR 45–140) for moderate COVID-19 and 132 mg/L (IQR 61–269) for severe COVID-19 (*p* < 0.001; Mann–Whitney U test). Receiver operating characteristic (ROC) curve analysis demonstrated a higher area under the curve (AUC) for calprotectin than CRP in the detection of severe COVID-19 (Figure 1).

Sensitivity, specificity, positive predictive values (PPVs), and negative predictive values (NPVs) for different cutoffs of calprotectin for severe COVID-19 are shown in Table S1 (Supplementary Materials). Using a cutoff of 4 mg/L, serum calprotectin distinguished severe from moderate disease with a sensitivity of 66%, specificity of 73%, PPVs of 54%, and NPVs of 82%. This cutoff of 4 mg/L was used to define the high calprotectin group in the proteomic analyses. Characteristics of patients with calprotectin ≥4 mg/L and <4 mg/L are shown in Table 1. The groups were comparable regarding baseline data, although the group with calprotectin ≥4 mg/L was more severely ill and was more likely to be treated with corticosteroids.

### 2.3. Longitudinal Changes in Serum Calprotectin

**Figure 2:**
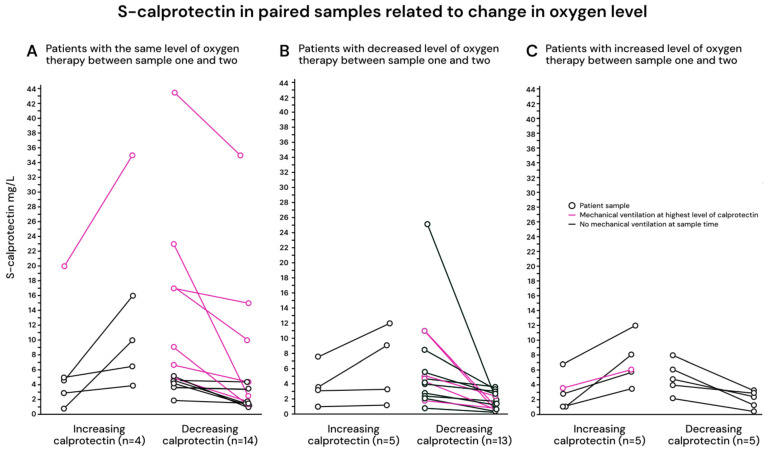
(**A**–**C**) Calprotectin changes in all patients with two samples analyzed, related to respiratory status. (**A**) Patients with stable respiration (the same level of oxygen between samples), (**B**) patients with improved respiration (decreased level of oxygen), and (**C**) patients with worsened respiration (increased level of oxygen).

In total, 46 patients (29%) had calprotectin analyses performed on two subsequent samples during the hospital stay. The median time between samples was 7 days (IQR 5–12 days). There were 14 patients with increasing calprotectin and 32 patients with decreasing calprotectin between samples. Figure 2A–C illustrates these patients stratified by respiratory status: (A) stable respiration, (B) improved respiration, i.e., decreased level of oxygen, and (C) worsened respiration, i.e., increased level of oxygen (C). Increasing calprotectin between samples was noted in 28% (5/18) with stable respiration, 22% (4/18) with improved respiration, and 50% (5/10) with worsened respiration (not statistically significant).

Table S2 (Supplementary Materials) shows characteristics of patients with increasing and decreasing calprotectin between samples. Patients with decreasing calprotectin had received corticosteroids before sample one and had severe COVID-19 on sampling day one significantly more often than those with increasing calprotectin. Figure S1 (Supplementary Materials) shows calprotectin values in samples one and two in patients with no change, an increase, and a decrease in oxygen supply. There was a significant difference in calprotectin (decrease) only for those with a decrease in oxygen supply, who also had a decrease in calprotectin. Accordingly, those with decreasing calprotectin between samples had a significant decrease in level of care between samples, 1 (7%) of patients with increasing calprotectin had decreased level of care, and 10 (31%) patients with decreasing calprotectin had a decreased level of care at sample two (Table S2; Supplementary Materials). Calprotectin in sample two was significantly lower among patients who had received corticosteroids before sample one (Figure S2; Table S2; Supplementary Materials), but not among those receiving corticosteroids between sample one and two.

### 2.4. Serum Calprotectin and Circulating Proteome

#### 2.4.1. Serum Calprotectin Related to Proteomics Characteristics

**Figure 3:**
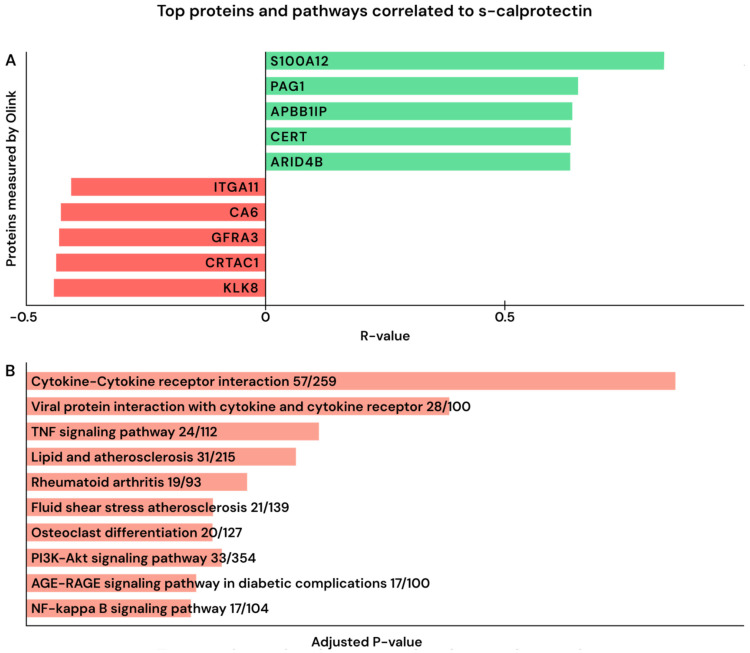
(**A**,**B**). (**A**) Correlation of calprotectin to proteins determined with the Olink Explore platform in sample 1. Displayed are the top 5 proteins that correlated significantly with calprotectin, both positively and negatively (FDR-adjusted *p* value < 0.05 calculated with Spearman correlation). (**B**) KEGG pathway analysis via Enrichr [38,39,40] of significantly positively correlating proteins measured with Olink Explore. The number after pathways indicates the number of overlapping proteins.

We then aimed to address the association of calprotectin with patterns in the circulating proteome. When correlating calprotectin values with all measured proteins (*n* = 1463), 563 (38%) proteins showed a significant positive correlation. In contrast, 136 (8%) proteins showed a significant negative correlation. The top 5 positively and top 5 negatively correlated proteins are shown in Figure 3A (the complete list of proteins analyzed with R-value, *p*-value, and adjusted FDR is provided in Excel File S1 Sheet 1, Supplementary Materials).

S100A12 stood out as the protein with the strongest correlation with calprotectin (r = 0.83 and FDR-adjusted *p*-value < 0.0001). Furthermore, P-antigen Family Member 1 (PAG1), Amyloid Beta Precursor Protein Binding Family B Member 1 Interacting Protein (APBB1IP), and Ceramide Transfer Protein Lipid Transfer Protein (CERT) were positively correlated with calprotectin levels. There were two other proteins from the S100A family that also significantly positively correlated to calprotectin, S100A11 and S100A16 (FDR adj. *p*-value < 0.0001 and 0.035). S100A4 was positively correlated, but this was not significant (FDR adj. *p*-value 0.26).

Pathway enrichment of positively correlated proteins using KEGG (Kyoto Encyclopedia of Genes and Genomes) [38,39,40] and all 1463 assayed targets as background revealed a strong over-representation of immune–inflammatory signaling. Top pathways included “Cytokine–cytokine receptor interaction (57/259)”, “Viral protein interaction with cytokine and cytokine receptor (28/100)”, “TNF signaling (24/112)”, and “NF-κB signaling (17/104)” (Figure 3B).

Kallikrein-8 (KLK8) and Cartilage Acid Protein 1(CRTAC1) were the proteins with the strongest negative correlations.

#### 2.4.2. Serum Proteomics Related to Serum Calprotectin Value and Disease Severity

**Figure 4:**
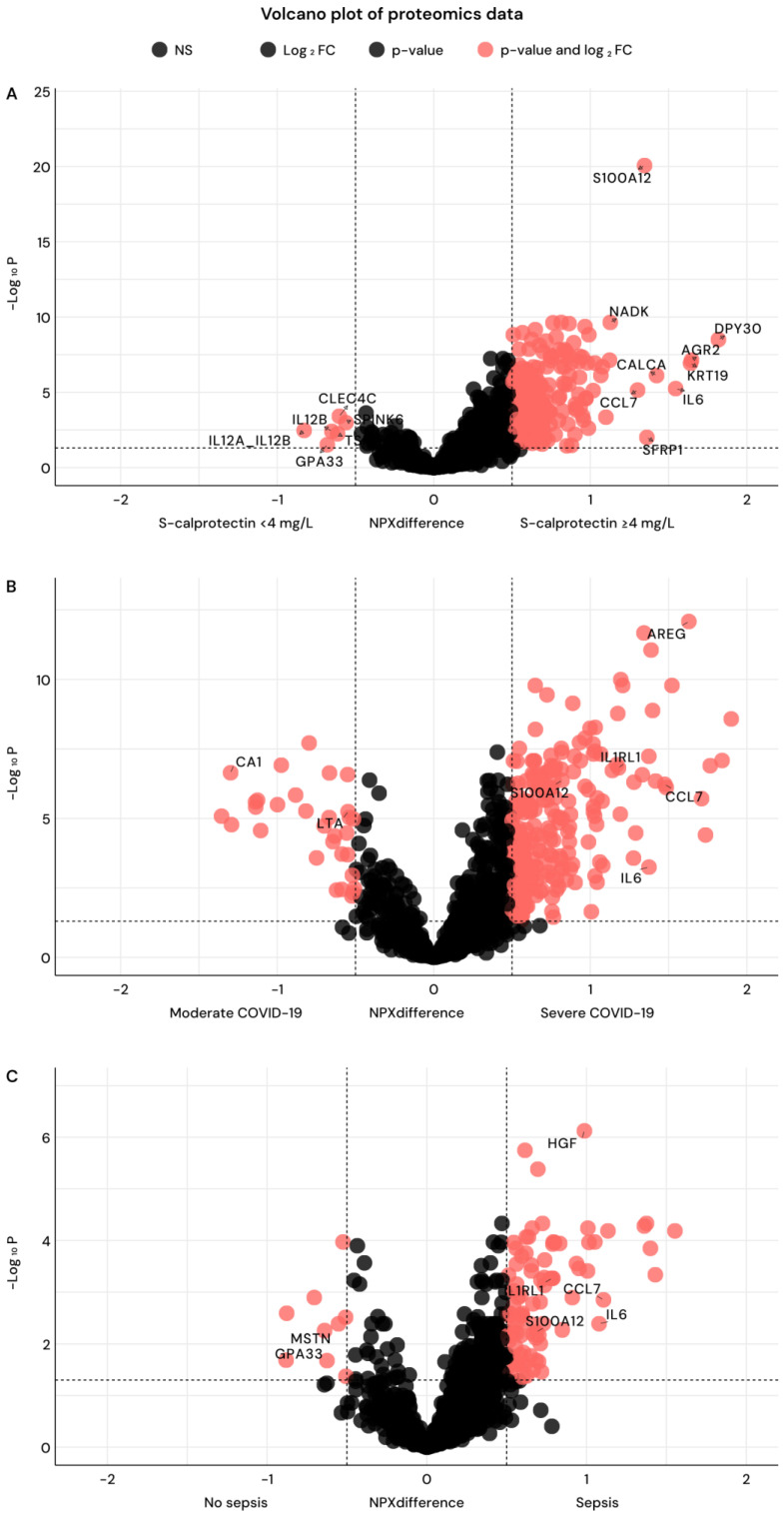
(**A**–**C**)**.** Volcano plot of proteomics data from the Olink Explore platform. The *x*-axis shows the difference in normalized protein expression (NPX) between compared groups, and the *y*-axis shows the −log10 (*p*-value) for each protein. Vertical dashed lines indicate NPX difference cut-offs at ±0.5 and the horizontal dashed line indicates (−log10(p) ≥ 1.3, corresponding to *p* < 0.05). All protein data included for each analysis are provided in Excel File S1; Supplementary Materials. (**A**) Comparison of patients with calprotectin ≥4 mg/L and <4 mg/L. (**B**) Comparison of patients with moderate and severe COVID-19. (**C**) Comparison of sepsis and non-sepsis COVID-19 patients.

We also compared the proteome between the patients with high calprotectin levels (≥4 mg/L) and those with low levels (<4 mg/L) (Figure 4A). Differential abundance analysis identified 187 (13%) proteins significantly increased in the high-calprotectin group (Figure 4A; Excel File S1, sheet 2 (Supplementary Materials)). The most prominent protein enriched in the high calprotectin group was S100A12. Other cytokines and immune mediators enriched in the high calprotectin group were CERT, NAD kinas (NADK), Protein Phosphatase 1 Regulatory Inhibitor Subunit 2 (PPP1R2), and APBB1IP.

When dividing patients into those with severe and moderate COVID-19, the most predominant protein was Amphiregulin (AREG); other significant proteins were IL6, Chemokine (C-C motif) ligand 7 (CCL7), and S100A12 (Figure 4B). We also compared all proteins in a volcano plot for COVID-19 patients divided into those with and without sepsis (Figure 4C). This was not surprisingly very similar to that of severe vs. moderate COVID-19. Among significant proteins were Hepatocyte Growth Factor (HGF), IL6, CCL7, and S100A12. All proteins for each plot are listed with NPX difference and *p*-value in Excel File S1; sheet 2 for high/low calprotectin; Excel File S1; sheet 3 for severe/moderate COVID-19, and Excel File S1; sheet 4 for sepsis/non-sepsis (Excel File S1 sheet 2, sheet 3, and 4; Supplementary Materials).

**Figure 5:**
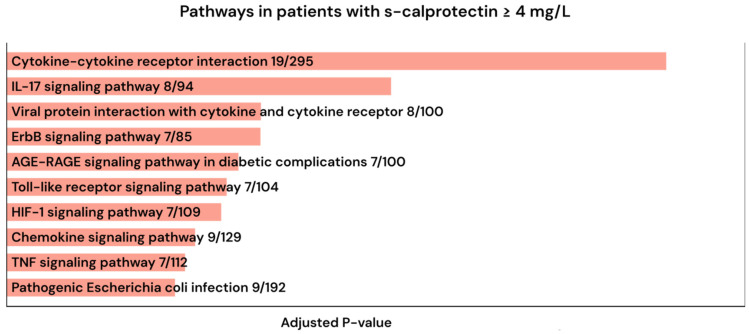
Pathway analysis of significantly elevated proteins in patients with calprotectin ≥ 4 mg/L using Enrichr [38,39,40].

Pathway analysis of proteins elevated in the calprotectin ≥ 4 mg/L group using KEGG revealed that these patients had a strong imprint of cytokine signaling and antiviral immune response as we found significant enrichment for KEGG pathways “cytokine-cytokine receptor interaction (19/295)”, “IL-17 signaling pathway (8/94)”, and “Viral protein interaction with cytokine and cytokine receptor (8/100)” (Figure 5).

KEGG pathways enriched using proteomics for severe COVID-19 were “Cytokine-cytokine receptor interaction” (63/295), “Viral protein interaction with cytokine and cytokine receptor” (26/100), and “PI3K-Akt signaling pathway” (40/354). The top ten pathways are displayed in Table S3 (Supplementary Materials). The top three KEGG-enriched pathways were identical for severe COVID-19 and COVID-19 with sepsis (Table S3, Supplementary Materials). The pathways were overall similar for calprotectin ≥4 mg/L, severe COVID-19, and COVID-19 with sepsis (Figure 4A–C and Figure 5 and Table S3, Supplementary Materials).

Proteins associated with calprotectin < 4 mg/L were Lymphotoxin-alpha (LT-α), Interleukin -12 (IL12), and C-Type Lectin Domain Family 4 Member C-(CLEC4C) (Figure 4A). However, KEGG enrichment showed only low signals in this group (Figure S3; Supplementary Materials).

#### 2.4.3. S100A12 and COVID-19

**Figure 6:**
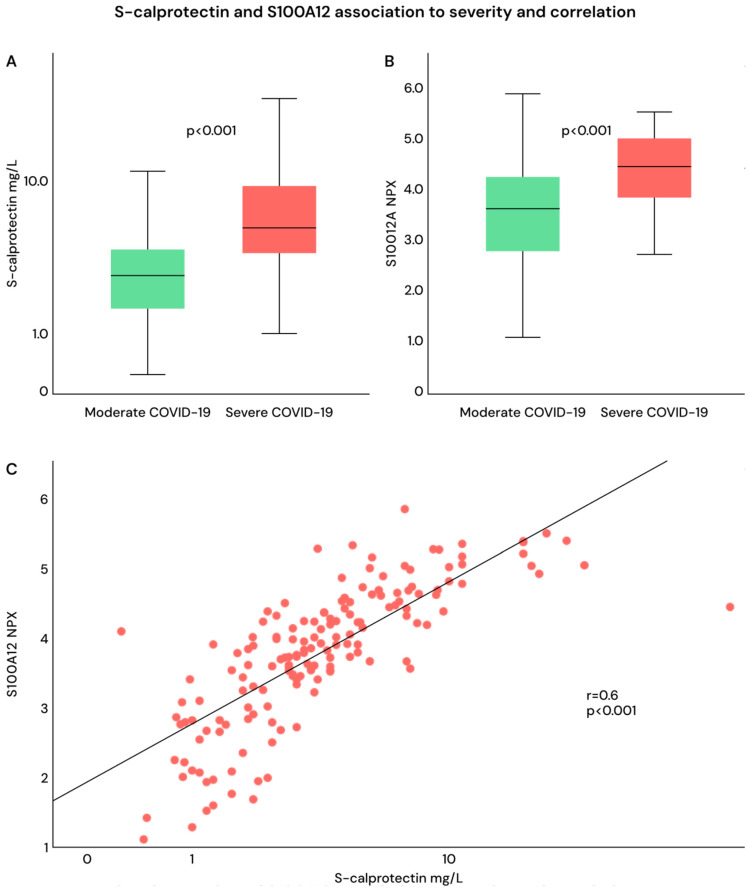
(**A**–**C**). (**A**,**B**) Calprotectin and S100A12 in patients with moderate and severe COVID-19. (**C**) Correlation between S100A12 and calprotectin, all patients.

Both S100A12 and calprotectin were significantly higher in patients with severe compared with moderate COVID-19 (Figure 6A,B), and they were significantly correlated with each other (Figure 6C).

We also compared S100A12 levels in the 160 study patients with those of 18 healthy controls (samples from the Karolinska KI/K COVID-19 biobank [37]) and found that the COVID-19 patients had significantly elevated S100A12 (Figure S4A; Supplementary Materials).

#### 2.4.4. Serum Calprotectin Dynamics Related to Serum Proteomics Changes

Changes in calprotectin levels over time in paired samples were correlated with longitudinal changes in the circulating proteome (Figure S5A; Supplementary Materials). Only minor changes in proteomic profiles were observed for both increasing and decreasing calprotectin levels. This was likely due to the low number of samples, and therefore, almost no proteins were significantly correlated. Only “cell adhesion molecules” were enriched with KEGG.

### 2.5. Serum Calprotectin Related to Neutrophil Activation

Since calprotectin is released from neutrophils, we wanted to explore the correlation between calprotectin and neutrophils. First, we correlated calprotectin with the absolute neutrophil count and noted a significant correlation (r = 0.2 and *p*-value < 0.001) (Figure S6A). To address the neutrophil function as well, we calculated a neutrophil activation score from the proteome data and correlated this with calprotectin levels. A significant correlation (r = 0.4; *p*-value < 0.0001) as well as a significantly elevated average neutrophil activation score was noted in patients with high calprotectin (*p* < 0.05) (Figure S6B,C; Supplementary Materials).

## 3. Discussion

In this observational study of hospitalized adults with COVID-19 as a model of viral sepsis, serum calprotectin was clearly associated with both disease severity and sepsis according to Sepsis-3 and outperformed CRP for identifying severe disease. To improve the knowledge gap regarding the biological interpretation of calprotectin, we also analyzed the blood proteome of the patients. We found that high calprotectin had a proteomic pattern similar to that of severe disease and was linked to a neutrophil-centric inflammatory proteomic signature, in particular, a close correlation with S100A12. This included upregulation of cytokine and danger-signaling pathways (TNF, NF-κB, IL-17; cytokine–receptor interactions) and a positive correlation with a neutrophil activation score. Together, these findings suggest that calprotectin reflects both current disease severity and the antiviral hyperinflammatory milieu.

Calprotectin has different cut-off values if measured in blood, plasma, or serum, but for serum, the reference limit is below 1.41 mg/L [41]. Using sensitivity and specificity analyses, we found that the cut-off of 4 mg/L could be relevant for discriminating between severe and non-severe COVID-19. Two previous studies report similar cut-offs, 4.7 mg/L and 3.0 mg/L, respectively [20,23]. No generally established cut-off for COVID-19 severity exists, and the cut-off of 4 mg/L was applied to stratify patients into a clinically meaningful group rather than for optimal discrimination. This cut-off was selected to ensure consistency with previously published studies, despite a moderate sensitivity (66%) for severe disease in our cohort. In our study, sepsis was noted in 87% of patients with calprotectin ≥4 mg/L and in 58% of those with calprotectin <4 mg/L.

S100A12 was in all our analyses the single protein with the strongest correlation to calprotectin, both overall and among patients with calprotectin of ≥4 mg/L. S100A12 is a close relative to calprotectin (S100A8/S100A9) and belongs to a sub-family also called Calgranulins or myeloid-related proteins [11]. S100A12 is present in the cytoplasm of neutrophils and on the plasma membrane of monocytes, similar to calprotectin [12,13]. Two other members of the S100A family were also significantly correlated with calprotectin (but not as strongly), and one was not.

Previous studies have reported elevated levels of S100A12 in COVID-19, with higher levels in severe compared with moderate disease [42]. Mester et al. also found high levels of S100A12 to be associated with reactivation of Herpes Simplex Virus and bacterial superinfection [43]. S100A12 has further been linked to community-acquired pneumonia (CAP) severity and outcome [44,45,46] and found to be a potential biomarker for sepsis [47]. Within the Human Protein Atlas project, S100A12 was highly expressed in acute viral and bacterial infections but not in outpatients with HIV; see Figure S6B (Supplementary Materials) [48].

The strong correlation between calprotectin and S100A12 suggests that these proteins might be released simultaneously or in response to the same inflammatory stimuli. We have previously shown that calprotectin is more useful than CRP for detecting sepsis requiring admission to intensive care [31], and in the present study, it was superior for detecting severe COVID-19. Given the close correlation and the ability of S100A12 to distinguish severe from moderate COVID-19, S100A12 may have similar diagnostic and prognostic properties.

Both S100A12 and calprotectin have been investigated in treatment-related studies. Tocilizumab has been shown to downregulate S100A12 expression [46], potentially contributing to its effect in severe COVID-19. Elevated plasma S100A8/A9 levels were also associated with left-ventricular systolic dysfunction in severely ill sepsis patients [49]. In a mouse model, pharmacological blockade of S100A8/A9 markedly reduced inflammation and mitigated sepsis-induced myocardial dysfunction. Thus, there may be a potential therapeutic role for targeting S100A8/A9, with calprotectin used as a biomarker to identify patients with potential benefit from such therapy.

Several additional proteins were correlated with calprotectin levels. APBB1IP, which plays a role in T-cell activation and regulates cell adhesion, was one such protein [50]. Given that COVID-19 was a newly emerging disease in 2020, T cells were critical for both viral clearance and the development of long-term immune memory [51]. CERT was also correlated with calprotectin; this protein has been linked to tumor cell death. As no clear association between COVID-19 and tumor suppression has been described, this finding was unexpected and of uncertain relevance [52,53].

Furthermore, NADK, protein dpy-30 (DPY30), calcitonin-related polypeptide alpha (CALCA), anterior gradient 2 (AGR2), and IL-6 were also correlated with calprotectin.

The proteomic pattern associated with calprotectin ≥4 mg/L was similar to that associated with severe COVID-19 and COVID-19 with sepsis. Proteins associated with COVID-19 severity included AREG, believed to play a role in cell repair [54], IL-1, a well-known cytokine involved in acute infection [55], CCL7, which recruits various leukocytes to mediate the immune response [56], HGF, which, among other functions, is involved in T-cell migration [57], and S100A12.

Pathway analyses indicated strong associations with cytokine-driven viral pathways, which is not unexpected given that all patients in this study were hospitalized due to COVID-19.

We observed the close association between neutrophils and calprotectin, both via direct correlation with neutrophil numbers and with the neutrophil activation score. This is of interest given the recent findings on the role of calprotectin during neutrophil activation [58]. There is no evidence regarding clinical significance but considering other publications showing the link between neutrophil activation and calprotectin function, this association warrants further investigation [58,59].

In repeated samples, calprotectin decreased with improved respiratory status and in patients who had received corticosteroid treatment prior to the first sample. The latter decrease was probably due to clinical improvement rather than a direct steroid effect, as calprotectin has been associated with disease progression independently of corticosteroid use [17].

In this study, COVID-19 was used as a model for viral sepsis. Viral sepsis is not common and is mostly associated with influenza [7]. Sepsis is defined as infection with organ dysfunction [60], but in the systematic review made by Karakike et al., only nine out of 151 included original COVID-19 studies mentioned the term sepsis [7]. Even though severe COVID-19 predominantly affects a single organ, it fulfills the criteria for sepsis. Sepsis and severe COVID-19 share features of extensive cytokine release, and our results show that at least one biomarker, calprotectin, is elevated in both conditions. Changes in the SARS-CoV-2 subtype and the immune status in the global population have made COVID-19 a less severe disease [61,62]. However, research on COVID-19 remains relevant as a model for viral sepsis. Calprotectin is a promising biomarker of disease severity and further research on its role in the pro-inflammatory cascade may contribute to a better understanding of sepsis in general.

The major strengths of the study include a prospective enrollment design with standardized sampling and the integration of calprotectin measurements with high-dimensional proteomics and pathway analyses.

The study has limitations, including the single-center design, sampling several days after admission, and the cohort-derived calprotectin cut-off, which requires external validation.

## 4. Materials and Methods

### 4.1. Patients and Clinical Data

This is an observational study of adult patients hospitalized for COVID-19 at Karolinska University Hospital, Stockholm, between April and October 2020 [37]. Inclusion criteria were hospitalized adult patients (over 18 years), positive PCR for SARS-CoV-2 on admission day, and blood samples collected for the study within 8 days of hospital admission. All samples were part of the Karolinska KI/K COVID-19 biobank [37].

Patient data (sex, age, comorbidity, oxygen supply, laboratory workup (including neutrophils, CRP, platelet count, bilirubin, and creatinine), blood cultures, vital parameters, ICU care, in-hospital mortality, and medical treatments (including corticosteroids, low molecular weight heparin, and remdesivir)) were manually extracted from the electronic health record system (Take Care).

### 4.2. Patient Classification

COVID-19 disease severity was defined retrospectively according to the level of respiratory support on the day of inclusion:

(i) Moderate COVID-19 is defined as requiring ≤10 L/min supplementary oxygen; and
(ii) Severe COVID-19 is defined as requiring >10 L/min supplementary oxygen, high-flow nasal oxygen (HFNO), non-invasive ventilation (NIV), or invasive mechanical ventilation.

This classification was consistent with clinical practice and has been used in previous studies from the Karolinska KI/K COVID-19 Study Group [14].

Sepsis was defined as a separate clinical condition using the SOFA score according to the Sepsis-3 definition [60], calculated on the day of inclusion.

### 4.3. Sample Collection

Patients were included and sampled once a week in the wards (infectious disease ward, ICU, and the wards for elderly people), but not in the emergency department. If possible, patients provided at least one sample for follow-up. The second sample was obtained at a median of 7 days from the first (IQR 5–14 days). Forty-six patients (28%) had two samples available for this study.

Blood samples were collected in serum tubes without gel. The tubes were centrifuged within 4 h, serum was aspirated, and serum aliquots were frozen at −80 °C until analysis.

Serum samples were also collected from 18 healthy controls during the time of patient inclusion and analyzed for proteomics, but these samples were not available for calprotectin analysis.

### 4.4. Calprotectin Assay

Serum calprotectin concentrations were measured using Gentian’s immunoturbidimetric Gentian GCAL (Gentian^®^ A/S, Moss, Norway) assay at Karolinska University Laboratory, Karolinska University Hospital, Huddinge, Sweden. This is currently a routine clinical test at Karolinska University Laboratory. Calibration was performed with the Gentian Calprotectin Calibrator Kit (Gentian^®^ A/S, Moss, Norway). Calibration was performed using a six-point calibration curve covering a measuring range of 0–21.25 mg/L.

All analyses were carried out on a Cobas c502 automated chemistry analyzer (Roche Diagnostics^®^, Mannheim, Germany), which is used for clinical routine testing. Samples were analyzed continuously and were not processed in batches.

Method imprecision was evaluated during the verification phase using low- and high-level control materials provided in the Gentian Calprotectin Calibrator Kit. Total imprecision, expressed as the coefficient of variation (CV%), was calculated for each control level based on 20 independent measurements, performed as four replicate measurements per day over five consecutive days. The total CV was 2.4% at a concentration of 10 mg/L and 6.2% at 1.0 mg/L. These results are consistent with published analytical performance data for the Gentian GCAL^®^ Calprotectin Immunoassay [63].

### 4.5. Method for Measuring Proteomics in Serum

Proteomic analysis was performed on serum samples from patients and healthy controls, with the Proximity Extension Assay technology via the Olink Explore panel ^®^ (Olink Explore 1536, Olink Proteomics, Uppsala, Sweden). The method is previously described, and the COVID-19 cohort is partly the same as in a previous paper [64]. In brief, 40 µL of serum was shipped to Olink^®^ and was analyzed with their proximity extension assay that detects proteins via oligonucleotide-linked antibodies and is a PCR-based method. Both analysis and quality control were performed by Olink. The results are in an arbitrary unit (NPX value) that allows relative quantification. Proteins failing quality control (*n* = 73) were excluded, and all remaining proteins (*n* = 1463) were included in the analysis.

### 4.6. The Neutrophil Activation Score

The neutrophil activation score was calculated by us, but we used a review by Tecchio et al. [59] as a reference for proteins that are important in relation to neutrophils and neutrophil activation. In detail, data on the following proteins that were described as relevant for neutrophils [59] was included from Olink analysis: CCL2, CCL3, CCL4, CCL17, CCL18, CCL19, CCL20, CCL22, CXCL1, CXCL3, CXCL5, CXCL6, CXCL8, CXCL9, CXCL10, CXCL11, CXCL12, CXCL13, CSF1, IL-1RA, TGFB1, TGFB2, IL1B, IL1A, IL6, IL7, IL12A_IL12B, IL18, EBI3_IL27, MIF, TSLP, VEGFA, HBEGF, FGF2, TGFA, HGF, ANGPT1, TNF, FASLG, CD40L, TNFSF11, CD257, TNFSF14, TNFSF13, OSM, AREG, MDK, EDN, and NGF. Next, z-scores were calculated for each protein. The individual sum of z-scores was then calculated for the individual neutrophil activation score.

### 4.7. Statistical Analysis

The correlation between calprotectin and proteomic data was calculated with Spearman’s correlation. Statistically significant comparisons between two groups in proteomic analysis were calculated with an unpaired *t*-test and FDR-adjusted *p*-values. Pathway analysis of proteins of interest was performed using Enrichr KEGG pathway [38,39,40,65]. SPSS version 28.0.0 was used for an independent *t*-test determination between calprotectin results. For analyses regarding proteomics and calprotectin, R version 4.4.2 was used, as well as GraphPad Prism 10.6.

### 4.8. Institutional Review Board Statement

The study was approved by the Swedish Ethical Review Authority (DNR 2020-01558, date 22 April 2020; DNR 2020-05069, date 6 October 2020).

## 5. Conclusions

The present study of hospitalized patients with COVID-19, used as a model for viral sepsis, found that high calprotectin was linked to disease severity and to a broad, neutrophil-centric inflammatory proteomic signature, encompassing cytokine and danger-signaling pathways, as well as an elevated neutrophil activation score. S100A12, the protein most strongly correlated with calprotectin, was also associated with disease severity. This study provides improved knowledge that can help in further studies regarding sepsis and COVID-19. Future studies should validate the link between calprotectin and the circulating proteome in other sepsis etiologies and explore whether S100A8/A9 (calprotectin)- and S100A12-related pathways could be targeted therapeutically.

## Figures and Tables

**Table 1:** Clinical characteristics of study patients in total and according to levels of calprotectin.

Characteristics	All Patients *n* = 160 No. (%) Unless Specified	Subgroup with Different Levels of Calprotectin		
		Calprotectin < 4 mg/L *n* = 91	Calprotectin ≥ 4 mg/L *n* = 69	*p*-Value
*Baseline characteristics*				
Sex, female	42 (26%)	26 (29%)	16 (23%)	0.44
Age median (IQR)	59 (51.0–67.0)	60 (61.5–74)	59 (56–69.5)	0.43
Charlson Comorbidity Index, median (IQR)	1 (0–2.5)	1 (0–2)	1 (0–3)	0.47
*Comorbidity before hospitalization included in CCI*				
Myocardial infarction	21 (13%)	12 (13%)	9 (13%)	0.98
Hart failure	13 (8%)	8 (9%)	5 (7%)	0.72
Peripheral vascular disease	5 (3%)	2 (2%)	3 (4%)	0.44
Hypertonia	74 (46%)	37 (41%)	37 (54%)	0.10
Stroke	7 (4%)	3 (3%)	4 (6%)	0.44
Dementia	7 (4%)	3 (3%)	4 (6%)	0.44
Chronic obstructive pulmonary disease	32 (20%)	18 (20%)	14 (20%)	0.94
Connective tissue disease	10 (6%)	6 (7%)	4 (6%)	0.84
Ulcer	1 (1%)	1 (1%)	0 (0%)	0.38
Diabetes mellitus	37 (23%)	19 (21%)	18 (26%)	0.44
Hemiplegia	1 (1%)	0 (0%)	1 (1%)	0.25
Kidney failure	8 (5%)	4 (4%)	4 (6%)	0.69
Liver failure	6 (3%)	3 (3%)	2(3%)	0.89
Malignancy	13 (8%)	8 (9%)	5 (7%)	0.72
AIDS	0 (0%)	0 (0%)	0 (0%)	0.92
Connective tissue disease	25 (16%)	14 (15%)	11 (16%)	0.78
Neurological disease	8 (5%)	4 (4%)	4 (6%)	0.69
Days from admission to sample one, median (IQR)	4 (3–6)	4 (2–5)	5 (3–6)	0.002
Days from first symptoms to sample, median (IQR)	13 (10–15)	12.5 (4.4) (10.0–15.0)	13 (5.4) (9–15)	0.61
Length of hospital stay in days, median (IQR)	10 (7–17)	8 (5–12)	14 (10–21)	<0.001
SOFA score, median (IQR)	2 (1–3)	2 (1–2)	2 (2–4)	<0.001
Sepsis, according to sepsis-3, at the first blood sample	113 (70%)	52 (57%)	61 (87%)	<0.001
Severe COVID-19 in the first blood sample	51 (32%)	14 (15%)	40 (58%)	<0.001
*Treatment before the first blood sample*				
Corticosteroids	69 (43%)	30 (33%)	39 (57%)	0.003
Cytokine-inhibitor	10 (6%)	3 (3%)	7 (10%)	0.08
Remdesivir	3 (2%)	2 (2%)	1 (1%)	0.73
Low molecular weight heparin	153 (96%)	87 (96%)	66 (96%)	0.99
*Oxygen supply at the first blood sample (%)*				
No oxygen	43 (27%)	34 (37%)	9 (13%)	<0.001
1–10 L/min of oxygen	63 (39%)	43 (47%)	20 (29%)	0.004
11–15 L/min of oxygen	3 (2%)	0 (0%)	3 (4%)	0.08
High nasal flow oxygen	23 (14%)	9 (10%)	14 (20%)	0.12
Non-invasive ventilation	5 (3%)	1 (1%)	4 (6%)	0.07
Invasive mechanical ventilation	23 (14%)	4 (4%)	19 (28%)	0.002
*Biochemistry at the first blood sample, median (IQR)*				
Calprotectin (mg/L)	3.5 (2.0–5.6)	2.3 (0.96) (1.3–3.0)	6.7 (8.9) (4.7–9.3)	<0.001
CRP (mg/L)	94 (51.5–173)	69 (39–105)	157 (81–275)	<0.001
Neutrophils 10^×9^	6.2 (4.0–9.3)	4.7 (2.3–6.9)	7.5 (4.1–10.6)	<0.001
*Events during hospital stay*				
In-hospital mortality	15 (9%)	4 (4%)	11 (16%)	0.013
Thrombosis during hospital stay	10 (6%)	4 (4%)	6 (9%)	0.267
Secondary infection before sample 1	9 (6%)	2 (2%)	7 (10%)	0.031
Secondary infection after sample 1	16 (10%)	3(3%)	13 (19%)	0.001

## Data Availability

The original contributions presented in this study are included in the article/Supplementary Materials. Further inquiries can be directed to the corresponding author(s).

## Supplementary Materials

The following supporting information can be downloaded at https://www.mdpi.com/article/10.3390/ijms27031243/s1.

## Abbreviations

The following abbreviations are used in this manuscript:

HFNO	High-flow nasal oxygen
NIV	Non-invasive ventilation
KEGG	Kyoto Encyclopedia of Genes and Genomes
IQR	Interquartile range
CCI	Charlson co-morbidity index
ROC	Receiver operating characteristic
AUC	Area under the curve
PPVs	Positive predictive values
NPVs	Negative predictive values
PAG1	P-antigen Family Member 1
AREG	Amphiregulin
CCL7	Chemokine (C-C motif) ligand 7
HGF	Hepatocyte Growth Factor
APBB1IP	Amyloid Beta Precursor Protein Binding Family B Member 1 Interacting Protein
CERT	Ceramide Transfer Protein Lipid Transfer Protein
KLK8	Kallekrein-8
CRTAC1	Cartilage Acid Protein 1
NADK	NAD kinas
PPP1R2	Protein Phosphatase 1 Regulatory Inhibitor Subunit 2
LT-α	Lymphotoxin-alpha
IL-2	Interleukine
CLEC4C	C-Type Lectin Domain Family 4 Member
CAP	Community-acquired pneumonia
DPY30	Protein dpy-30
CALCA	Calcitonin Related Polypeptide Alpha
AGR2	Anterior gradient 2
OR	Odds ratio
CI	Confidence interval
