# Supplementary material for: Serum Calprotectin in Hospitalized Patients with COVID-19 in Relation to High-Dimensional Serum Proteomic Patterns
Source: Int J Mol Sci. 2026 Jan 26;27(3):1243. doi: 10.3390/ijms27031243 (PMC12898007; doi:10.3390/ijms27031243)
Supplement: Supplementary file 1 [file ijms-27-01243-s001.zip › Supplementary Material.pdf]

# Serum calprotectin in hospitalized patients with COVID-19, related to patterns in high-dimensional serum proteomics

Åsa Parke[*], Benedikt Strunt, Puran Chen, Karolinska KI/K COVID-19 Study Group, Dorota Religa, Hans-Gustaf Ljunggren, Olav Rooyackers, Soo Aleman, Anna Norrby-Teglund, Niklas K Björkström, Magnus Hansson, Kristoffer Strålin

## Supplementary material:

**Table S1**. Sensitivities, specificities, positive predictive values (PPV), and negative predictive values (NPV) of different cut-offs of s-calprotectin for detection of severe COVID-19

**Table S2.** Characteristics of patients with increasing and decreasing s-calprotectin

**Tabel S3.** Showing Enriched pathways with overlap, p-value and adjusted p-value for patients with severe COVID-19 and COVID-19 patients with sepsis.

**Figure S1.** Patients paired samples analyzed, divided in those with the same level of oxygen between samples, increased level of oxygen and decreased level of oxygen between samples

**Figure S2.** S-calprotectin related to corticosteroids therapy in patients with paired samples analyzed.

**Figure S3.** Pathway analysis via Enrichr of significantly enriched proteins in patients with calprotectin below 4 mg/L.

**Figure S4 A-B.** A) S100A12 in healthy controls and our patient cohort B) Data from Human Protein Atlas S10012A in healthy controls and other infections (not COVID-19).

**Figure S5 A-B.** Volcano plot displaying differentially enriched proteins (determined with Olink Explore platform) in patients with consecutive s-calprotectin samples. Difference between patients with A) increasing and B) decreasing s-calprotectin.

**Figure S6 A-C.** A) Correlation of neutrophil count and Calprotectin at sample 1. B) and C) display the relation of neutrophil activation score to calprotectin, displayed are both correlation (B), calculated with spearman correlation and groupwise comparison (C). P-value calculated with unpaired t-test.

**Excel File S1.** Sheet 1) Correlation of proteins determined with Olink Explore to calprotectin levels. Sheet2) Proteins determined in patient with severe vs moderate COIVD-19. Sheet 3) Proteins determined in patients with sepsis or not. Sheet 4) Proteins to calprotectin-levels in patients with high calprotectin >= 4mg/L.

**Table S1**. Sensitivities, specificities, positive predictive values (PPV), and negative predictive values (NPV) of different cut-offs of s-calprotectin for detection of severe COVID-19

| **S-calprotectin cut-off value (mg/L)** | **Sensitivity* %** | **Specificity** %** | **PPV %*** ** | **NPV %******* |
|---|---|---|---|---|
| 1 | 98 (50/51) | 11 (13/109) | 35 (50/146) | 93 (13/14) |
| 1.5 | 98 (50/51) | 23 (25/109) | 37 (50/134) | 96 (25/26) |
| 2 | 92 (47/51) | 35 (38/109) | 40 (47/118) | *90 (38/42)* |
| 2.5 | 90 (46/51) | 45 (49/109) | 43 (46/106) | 90 (49/54) |
| 3 | 82 (42/51) | 56 (62/109) | 47 (42/89) | 87 (62/71) |
| 3.5 | 76 (39/51) | 66 (72/109) | 51 (39/76) | 85 (72/84) |
| **4** | **66 (34/51)** | **73 (80/109)** | **54 (34/63)** | **82 (80/97)** |
| 4.5 | 60 (31/51) | 78 (85/109) | 56 (31/55) | 80 (85/105) |
| 5 | 50 (26/51) | 81 (89/109) | 56 (26/46) | 78 (89/114) |
| 5.5 | 49 (25/51) | 85 (93/109) | 60 (25/41) | 78 (93/119) |
| 6 | 43 (22/51) | 86 (94/109) | 60 (22/37) | 76 (94/123) |

*% (No. of true-positive cases/ all severe cases)
**% (No. of true-negative cases/ all moderate cases)
***% (No. of true-positive cases/ all cases over cut-off)
****% (No. of true-negative cases/ all cases under cut-off

**Table S2.** Characteristics of patients with increasing and decreasing s-calprotectin

| *Characteristic* | *Calprotectin increased (n=14)* | *Calprotectin decreased (n=32)* | *p-value* |
|---|---|---|---|
| Sex, female | 2 (14 %) | 9 (28%) | 0.40 |
| Age, median IQR | 65.5 (61.5-74) | 61 (56-69.5) | 0.317 |
| Charlson Comorbidity Index, median (IQR) | 2 (1-5.5) | 2 (0-3) | 0.327 |
| *Baseline data* | | | |
| Corticosteroids before sample | 3 (21%) | 24 (75%) | <0.001 |
| Cytokine inhibitor before sample | 1 (7%) | 4 (12%) | 0.595 |
| Remdesivir before sample | 1 (7%) | 0 (0%) | 0.131 |
| Days from admission to sample 1, median | 4 (3-5) | 5 (3-6) | 0.221 |
| Length of hospital stay in days, median | 19 (16.8-28.5) | 20 (14-41.5) | 0.774 |
| *Biochemistry (median IQR)* | | | |
| Calprotectin (mg/L) sample 1 | 3.4 (1.1-5.5) | 4.8 (3.6-8.8) | 0.037 |
| Calprotectin (mg/L) sample 2 | 6.3 (3.5-12) | 2.4 (1.3-3.56) | 0.002 |
| CRP (mg/L) sample 1 | 117 (47-227) | 130 (70-270) | 0.417 |
| CRP (mg/L) sample 2 | 107 (32-179) | 58 (12-143) | 0.174 |
| *Change in-between samples (%)* | | | |
| Increased level of care * | 1 (7%) | 1 (3%) | 0.417 |
| Decreased level of care * | 1 (7%) | 10 (31%) | 0.011 |
| Increased level of oxygen supply | 5 (36%) | 5 (16%) | 0.071 |
| Decreased level of oxygen supply | 4 (28%) | 14 (44%) | 0.059 |
| Secondary infection before sample 1 | 0 (0%) | 7 (22%) | 0.241 |
| Secondary infection between samples | 2 (14%) | 4 (12%) | 0.870 |
| Secondary infection after sample 2 | 0 (0%) | 7 (22%) | 0.060 |
| Severe COVID-19 at sample 1 | 5 (36%) | 26 (81%) | 0.006 |
| Severe COVID-19 at sample 2 | 7 (50%) | 13 (41%) | 0.956 |
| *Outcome data* | | | |
| In-hospital mortality | 1 (7%) | 6 (19%) | 0.319 |

*Different level of care was defined as i) regular ward (low level) ii) High dependency unit (medium level) iii) Intensive care unit (high level).

**Tabel S3.** Enriched pathways with overlap, p-value and adjusted p-value for patients with severe COVID-19 and COVID-19 patients with sepsis.

| Patients with severe COVID-19 | | | | Patients with COVID-19 and sepsis | | | |
|---|---|---|---|---|---|---|---|
| Pathway | Overlap | p-value | Adjusted p-value | Pathway | Overlap | p-value | Adjusted p-value |
| Cytokine-cytokine receptor interaction | 63/295 | 5.3E-39 | 1.4E-36 | Cytokine-cytokine receptor interaction | 26/295 | 3.7E-15 | 8.6E-13 |
| Viral protein interaction with cytokine and cytokine receptor | 26/100 | 5.4E-19 | 7.0E-17 | Viral protein interaction with cytokine and cytokine receptor | 14/100 | 2.0E-11 | 2.3E-9 |
| PI3K-Akt signaling pathway | 40/354 | 8.7E-15 | 7.6E-13 | PI3K-Akt signaling pathway | 19/354 | 8.4E-8 | 6.0E-6 |
| Rheumatoid arthritis | 20/93 | 3.3E-13 | 2.16E-11 | ECM-receptor interaction | 10/88 | 1.2E-7 | 6.0E-6 |
| MAPK signaling pathway | 32/294 | 1.2E-11 | 6.4E-10 | TNF signaling pathway | 11/112 | 1.3E-7 | 6.0E-6 |
| ECM-receptor interaction | 17/88 | 1.2E-10 | 5.29E-9 | Focal adhesion | 14/201 | 2.0E-7 | 6.9E-6 |
| Cell adhesion molecules | 21/148 | 3.4E-10 | 1.2E-8 | Rheumatoid arthritis | 10/93 | 2.1E-7 | 6.9E-6 |
| Malaria | 13/50 | 3.7E-10 | 1.21E-8 | Lipid and atherosclerosis | 14/215 | 4.5E-7 | 1.3E-5 |
| Lipid and atherosclerosis | 25/215 | 5.6E-10 | 1.6E-8 | MAPK signaling pathway | 16/294 | 7.5E-7 | 1.9E-5 |
| Fluid shear stress and atherosclerosis | 20/139 | 7.1E-10 | 1.7E-8 | Proteoglycans in cancer | 13/205 | 1.6E-6 | 3.6E-5 |

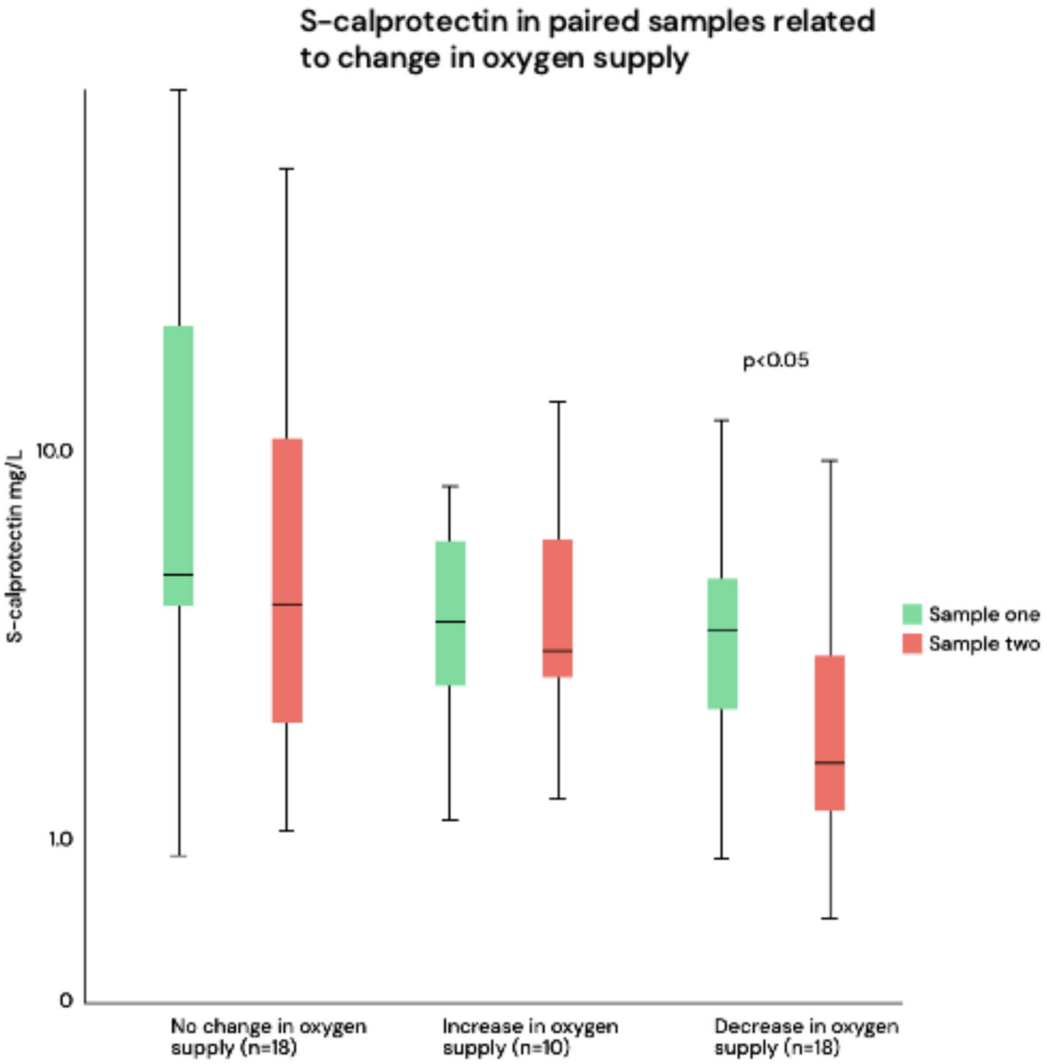


**Figure S1.** Patients paired samples analysed, divided in those with the same level of oxygen between samples, increased level of oxygen and decreased level of oxygen between samples.

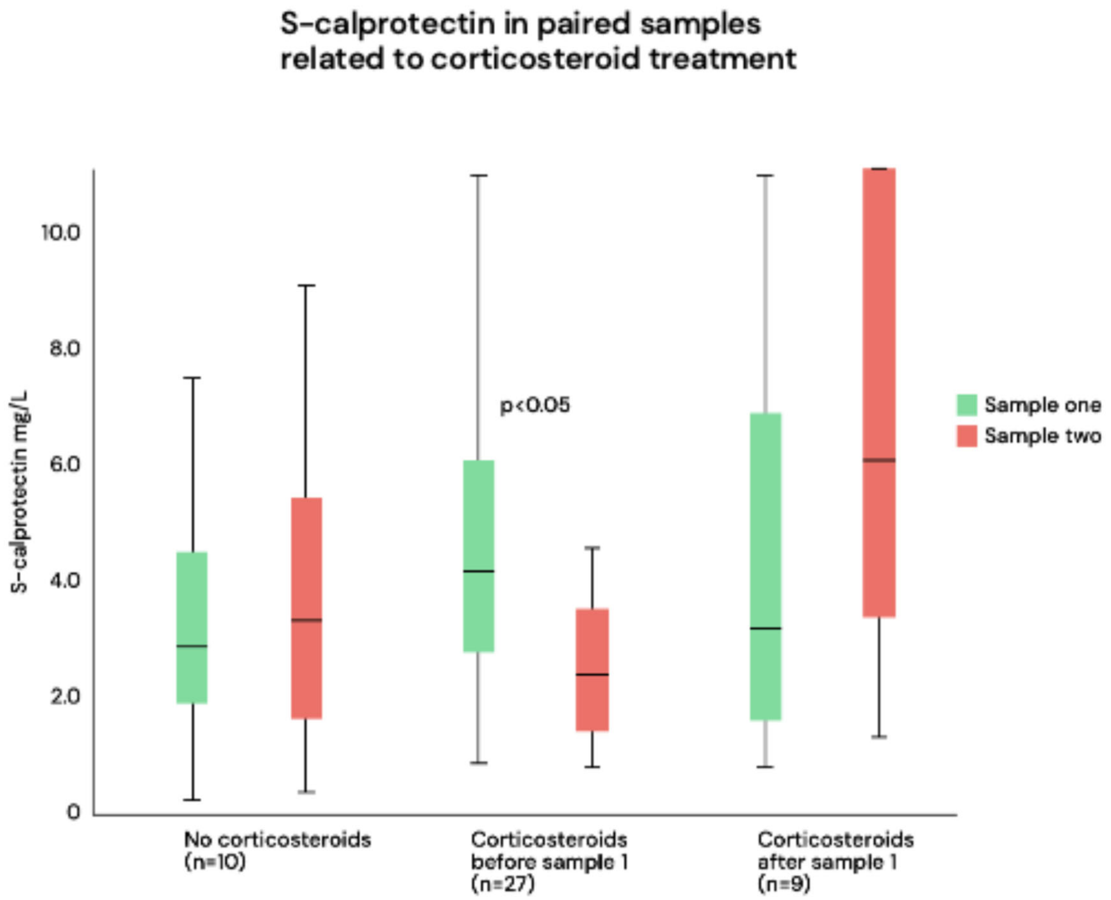


**Figure S2.** S-calprotectin related to corticosteroids therapy in patients with paired samples analysed.

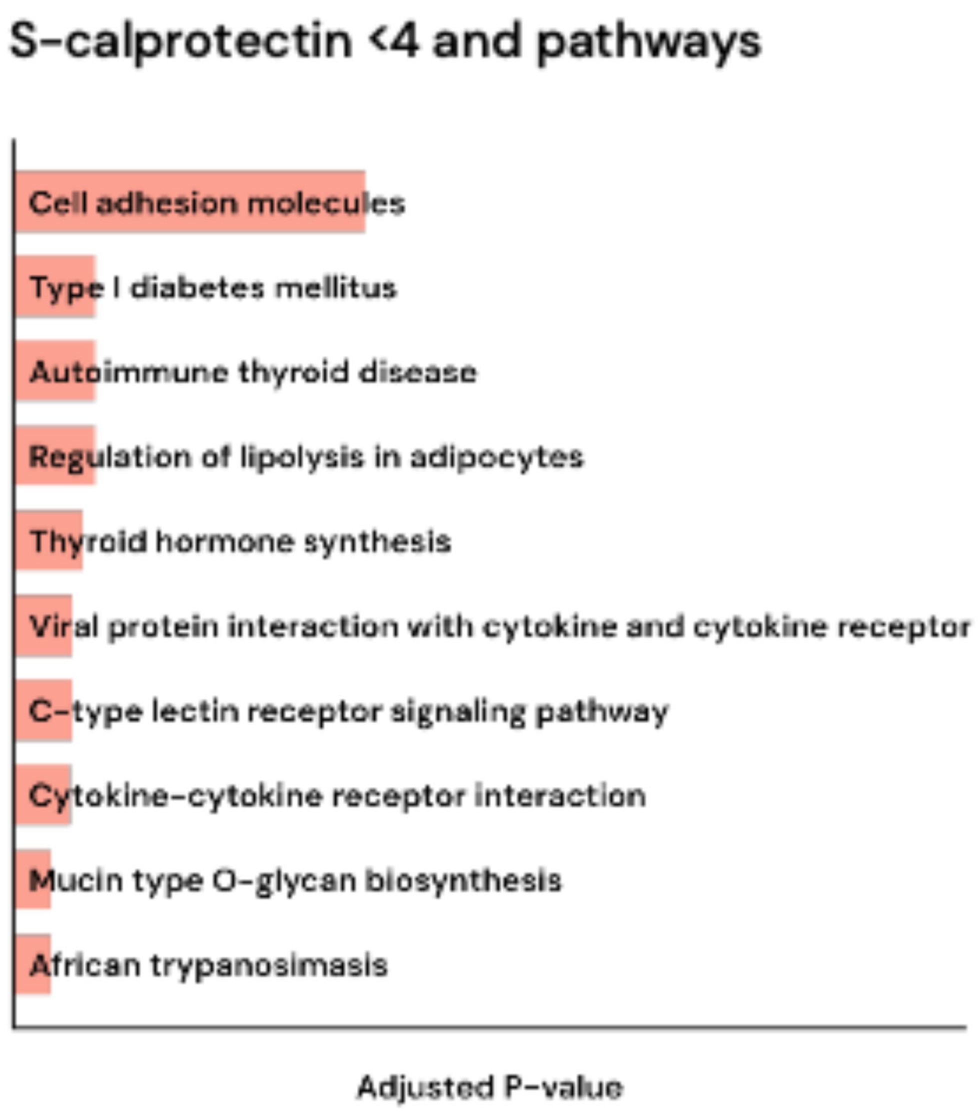


**Figure S3.** Pathway analysis via Enrichr of significantly enriched proteins in patients with calprotectin below 4 mg/L.

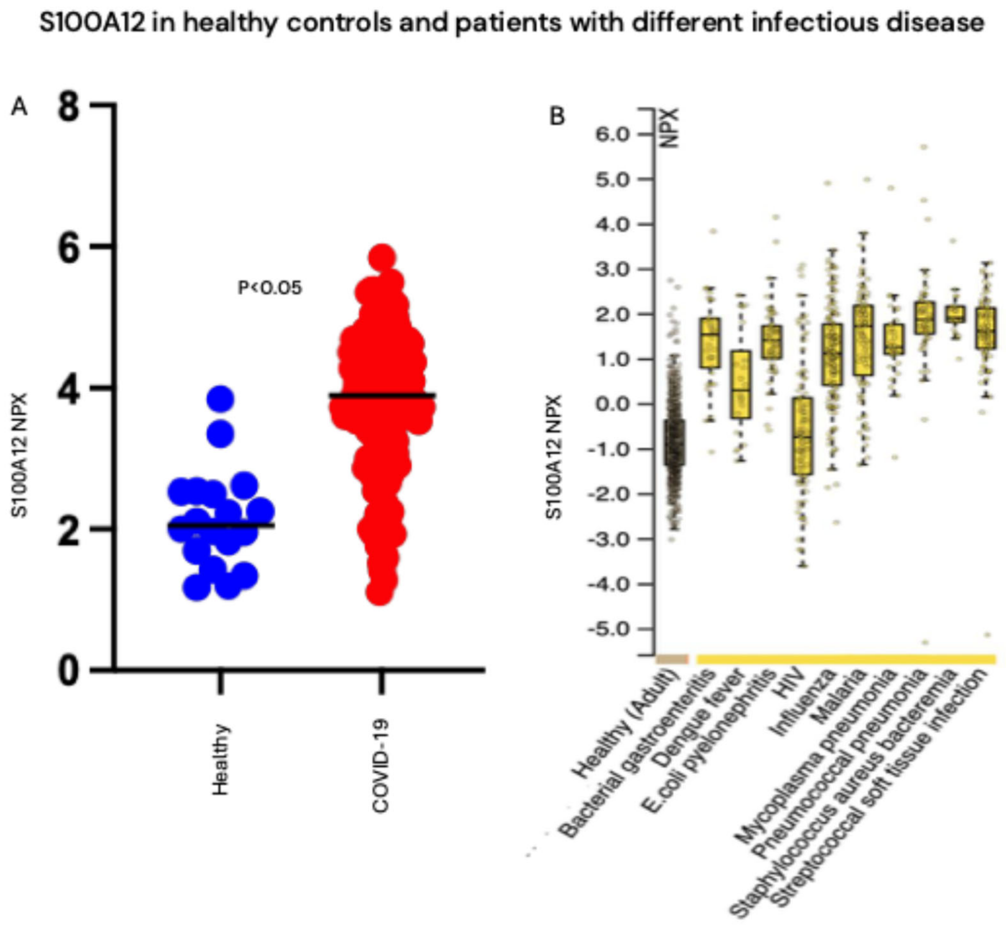


**Figure S4 A-B.** A) S100A12 in healthy controls and our patient cohort B) Data from Human Protein Atlas S10012A in healthy controls and other infections (not COVID-19). The graph was prepared by Fredrik Edfors at SciLifeLab, Stockholm, Sweden.

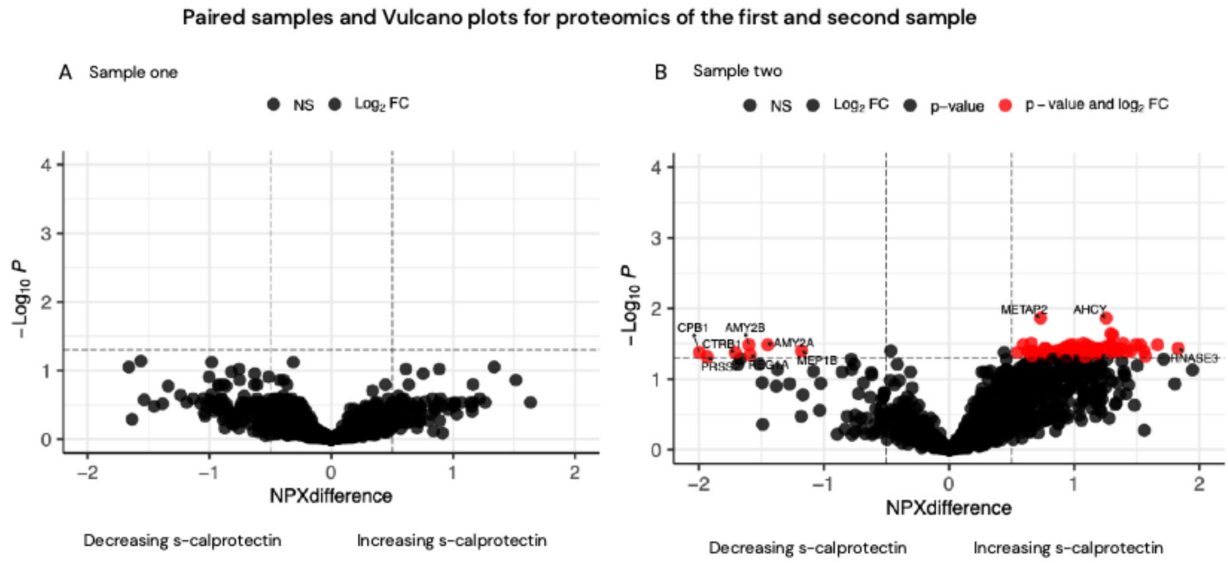


**Figure S5 A-B.** Volcano plot displaying differentially enriched proteins (determined with Olink Explore platform) in patients with consecutive s-calprotectin samples. Difference between patients with A) increasing and B) decreasing s-calprotectin.

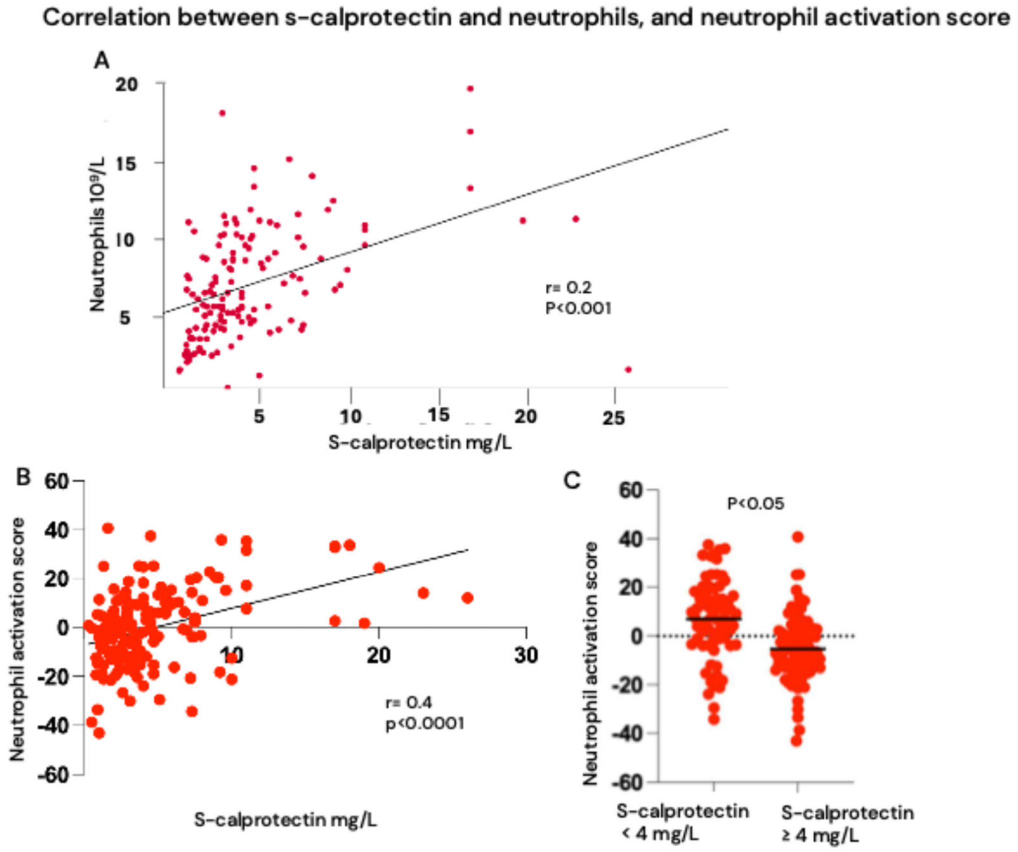


**Figure S6 A-C.** A) Correlation of neutrophil count and Calprotectin at sample 1. B) and C) display the relation of neutrophil activation score to calprotectin, displayed are both correlation (B), calculated with spearman correlation and groupwise comparison (C). P-value calculated with unpaired t-test.

**Excel-file S1.** Sheet 1) Correlation of proteins determined with Olink Explore to calprotectin levels. Sheet2) Correlation of proteins to calprotectin-levels in patients with high calprotectin >= 4mg/L. (supplied separately)
